# Role of Kallikrein 7 in Body Weight and Fat Mass Regulation

**DOI:** 10.3390/biomedicines9020131

**Published:** 2021-01-29

**Authors:** Anne Kunath, Juliane Weiner, Kerstin Krause, Maren Rehders, Anastasija Pejkovska, Martin Gericke, Martin L. Biniossek, Sebastian Dommel, Matthias Kern, Aleix Ribas-Latre, Oliver Schilling, Klaudia Brix, Michael Stumvoll, Nora Klöting, John T. Heiker, Matthias Blüher

**Affiliations:** 1 Medical Department III-Endocrinology, Nephrology and Rheumatology, University of Leipzig, 04103 Leipzig, Germany; kunath.anne@gmx.de (A.K.); juliane.weiner@medizin.uni-leipzig.de (J.W.); kerstin.krause@medizin.uni-leipzig.de (K.K.); sebastian.dommel@medizin.uni-leipzig.de (S.D.); matthias.kern@helmholtz-muenchen.de (M.K.); michael.stumvoll@medizin.uni-leipzig.de (M.S.); nora.kloeting@medizin.uni-leipzig.de (N.K.); 2 German Center for Diabetes Research (DZD), 85764 Neuherberg, Germany; 3 Department of Life Sciences and Chemistry, Jacobs University Bremen, 28759 Bremen, Germany; m.rehders@jacobs-university.de (M.R.); anja.pejkovska@gmail.com (A.P.); k.brix@jacobs-university.de (K.B.); 4 Institute of Anatomy and Cell Biology, Martin-Luther-University Halle-Wittenberg, 06108 Halle (Saale), Germany; martin.gericke@medizin.uni-halle.de; 5 Institute of Molecular Medicine and Cell Research, Faculty of Medicine, University of Freiburg, 79104 Freiburg, Germany; martin.biniossek@mol-med.uni-freiburg.de; 6 Helmholtz Institute for Metabolic, Obesity and Vascular Research (HI-MAG) of the Helmholtz Zentrum München at the University of Leipzig and University Hospital Leipzig, Philipp-Rosenthal-Str. 27, 04103 Leipzig, Germany; aleix.ribas@helmholtz-muenchen.de; 7 Medical Center, Institute of Surgical Pathology, Faculty of Medicine, University of Freiburg, 79106 Freiburg, Germany; oliver.schilling@mol-med.uni-freiburg.de; 8 Integrated Research and Treatment Center (IFB) AdiposityDiseases, University of Leipzig, 04103 Leipzig, Germany

**Keywords:** protease, inhibitor, adipose tissue, obesity, metabolic disease, treatment, kallikrein-related peptidase 7, KLK7

## Abstract

Increased plasma and adipose tissue protease activity is observed in patients with type 2 diabetes and obesity. It has been proposed that specific proteases contribute to the link between obesity, adipose tissue inflammation and metabolic diseases. We have recently shown that ablation of the serine protease kallikrein-related peptidase 7 (*Klk7*) specifically in adipose tissue preserves systemic insulin sensitivity and protects mice from obesity-related AT inflammation. Here, we investigated whether whole body *Klk7* knockout (*Klk7^−/−^*) mice develop a phenotype distinct from that caused by reduced *Klk7* expression in adipose tissue. Compared to littermate controls, *Klk7^−/−^* mice gain less body weight and fat mass both under chow and high fat diet (HFD) feeding, are hyper-responsive to exogenous insulin and exhibit preserved adipose tissue function due to adipocyte hyperplasia and lower inflammation. *Klk7^−/−^* mice exhibit increased adipose tissue thermogenesis, which is not related to altered thyroid function. These data strengthen our recently proposed role of *Klk7* in the regulation of body weight, energy metabolism, and obesity-associated adipose tissue dysfunction. The protective effects of *Klk7* deficiency in obesity are likely linked to a significant limitation of adipocyte hypertrophy. In conclusion, our data indicate potential application of specific KLK7 inhibitors to regulate KLK7 activity in the development of obesity and counteract obesity-associated inflammation and metabolic diseases.

## 1. Introduction

Obesity increases the risk to develop type 2 diabetes, cardiovascular and fatty liver diseases, likely in the context of promoting an underlying pro-inflammatory state [1]. Adipose tissue (AT) dysfunction is characterized by immune cell infiltration, adipocyte hypertrophy, ectopic fat deposition and altered adipokine secretion [2,3].

Elevated plasma and AT protease activity is associated with obesity, type 2 diabetes and chronic inflammation [4,5]. Intra- and extracellular proteases are significantly contributing to AT function, and dysregulation of proteolytic activities represents a direct link between obesity, AT inflammation and cardio-metabolic diseases. Global or conditional knock out of proteases or modulation of specific proteases’ activity reduced weight gain, inflammation and improved metabolic parameters under high fat diet (HFD) [5,6,7,8]. In accordance with that, overexpression of endogenous serpin inhibitors such as α1-antitrypsin (SERPINA1) and vaspin (SERPINA12) in AT reduces weight gain, inflammation and preserves insulin sensitivity under high fat diet [6,9].

In various mouse models, vaspin treatment improved glucose metabolism and reduced food intake [10,11,12,13]. Vaspin-mediated protection of AT from obesity-induced inflammation may contribute to improved insulin sensitivity, as has been shown in adipocytes in vitro [14]. Vaspin targets the serine protease KLK7 and the regulation of proteolytic activity represents an integral component of vaspins’ protective effects in obesity [15]. *Klk7* ablation in AT partially protects mice from HFD-induced epigonadal fat expansion, AT inflammation, and preserves systemic insulin sensitivity [16]. Moreover, *KLK7* was one of the most significantly upregulated genes during differentiation of human adipocytes from both lean and obese patients that may link inflammation and adipogenesis [17].

KLK7 is a member of the tissue kallikrein family that comprises 15 chymotrypsin- or trypsin-like serine proteases in humans [18,19]. KLK7 is predominantly expressed in the skin where it is involved in the desquamation process [20]. Aberrant KLK7 activity is a major underlying pathogenic mechanism of inflammatory skin diseases such as psoriasis [21], acne rosacea [22] and Netherton syndrome [23]. Quantitative mass spectrometry-based proteomics data shows highest KLK7 protein expression in hair follicles, the colon and esophagus [24]. KLK7 is also associated with tumor progression in various cancer types [25] and may therefore be considered a treatment target for a variety of diseases including those related to obesity [15,16].

Here, we test the hypothesis that whole-body ablation of *Klk7* cause a metabolically healthy phenotype that is distinct from that observed in mice with reduced *Klk7* AT expression.

## 2. Research Design and Methods

### 2.1. Animal Studies

Mice were housed in pathogen-free facilities (three to five mice per group and cage) at 22 ± 2 °C on a 12 h light/dark cycle. All mice had ad libitum access to water and food and were either fed a standard chow diet (EV153, 3.3% from fat, Ssniff^®^, Soest, Germany) or kept on a HFD containing 55% kJ from fat (E15772-34, Ssniff^®^, Soest, Germany) starting at an age of six weeks. 

### 2.2. Generation and Genotyping of Klk7^−/−^ Mice

Ablation of the *Klk7* gene was achieved by cross breeding previously described [16] floxed *Klk7^fl/fl^* mice (generated by Taconic-Artemis, Cologne, Germany) with mice ubiquitously expressing Cre-recombinase under the transcriptional control of a human cytomegalovirus (CMV) minimal promoter [26] (Jackson Laboratories, B6.C-Tg(CMV-Cre)1 Cgn/J, stock no. 006054). Recombination results in ubiquitous deletion of exons two to five with loss of function for the *Klk7* gene. Resulting *CMV-Cre^+^_Klk7^fl/fl^* mice (termed as *Klk7^−/−^* throughout the manuscript) were backcrossed on C57BL/6N background for at least five generations. Littermates without floxed *Klk7* gene (*CMV-Cre^+^_Klk7^+/+^*) or without the *Cre* gene (*CMV-Cre^−^_Klk7^fl/fl^*) were used as controls. Genomic DNA was isolated from the tail tip by using the DNeasy Kit (Qiagen, Hilden, Germany). Genotyping was performed by PCR using the following two primers (loxP site): 5′-GGGATGTAGGATTATGAGTGAGC-3′ (forward) and 5′-CAGTCCAGTGAACTGCTCACC-3′ (reverse); CMV-Cre: 5′-GCGGTCTGGCAGTAAAAACTATC-3′ (forward) and 5′-GTGAAACAGCATTGCTGTCACTT-3′ (reverse). DNA from control mice produced a 276 bp band, *Klk7^+/fl^* or *Klk7^fl/fl^* mice a 402 bp. Adipose-specific *Klk7^−/−^* knockout mice were previously described [16].

### 2.3. Phenotypic Characterization

Twelve male *Klk7^−/−^* mice and control littermates were studied from the age of six up to 32 weeks on chow diet. Total body weight was recorded weekly up to an age of 14 weeks, and thereafter in two-week intervals. Body composition, intraperitoneal (i.p.) insulin tolerance tests (ITT), glucose tolerance tests (GTT), indirect calorimetry were performed as described [16]. GTTs were performed after an overnight fast, ITT in ad libitum-fed mice. Under chow diet, GTT and ITT were performed at the age of 12 and 24 weeks, HFD animals underwent GTT and ITT at 18 weeks of age.

Blood glucose was determined from whole-venous blood samples using an automated glucose monitor (FreeStyle Mini; Abbott GmbH, Ludwigshafen, Germany). Serum adiponectin, chemerin, insulin, leptin, MCP-1, vaspin, fT3 and TT4 were measured by standard ELISAs [16]. Thyroid stimulating hormone (TSH) was determined in blood serum using a TSH-specific ELISA kit (CEA463MU, Cloud-Clone Corp., Houston, TX, USA). Serum concentrations of triglycerides (TGs), free fatty acids (FFAs) and cholesterol were analyzed by an automated system (COBAS 7000, Roche, Mannheim, Germany). For assessment of glycogen levels, liver tissues were homogenized and a glycogen assay kit was used (Biovision, Glycogen Assay Kit, Ilmenau, Germany).

Body composition was determined in conscious mice by nuclear magnetic resonance technology using an EchoMRI-700 instrument (Echo Medical Systems, Houston, TX, USA) in 10, 20 and 30 weeks old mice. Energy metabolism was analyzed using indirect calorimetry and CaloSys V2.1 metabolic chambers (TSE Systems, Bad Homburg, Germany) at an age of 30 weeks as previously described [16]. After 8–10 h of acclimatization, food and water intake, mean oxygen consumption (O_2_), CO_2_ production, respiratory exchange ratio, energy expenditure, spontaneous locomotor activity (X, Y, Z cage movement) and the distance run on a treadmill were recorded every 30 min for a total of 60 h. Rectal body temperature and body length (naso-anal length) were measured at the end of the study.

After sacrificing, organs were immediately removed, weighed (liver, subcutaneous inguinal (iWAT), epididymal (eWAT) and brown adipose tissue (BAT)) and frozen in liquid nitrogen. Measurements of adipocyte size distribution, and AT immunohistology was performed as described previously [16] using anti-CD16/32 (1:100; eBioscience, San Diego, CA, USA) to block Fc-receptors and stained by anti-CD45-e780 (1:200), anti-F4/80-PE-Cy7, anti-CD11c-PE, (all 1:100; eBioscience) and anti-CD206-Alexa647 (1:50; AbD Serotec, Kidlington, UK). DNA staining was performed using 7-aminoactinomycin D (7-AAD; BD Pharmingen, Feldkirchen, Germany).

### 2.4. RNA Isolation and Quantitative Real-Time PCR Analysis

RNA isolation and quantitative real-time PCR were performed as previously described [16]. All primer sequences are listed in Supplementary Table S1. Specific mRNA expression was calculated relative to *36B4* or *18S* using the ΔΔCT method, as indicated.

### 2.5. Primary Adipocyte Cell Culture and Western Blot Analyses

AT stromal vascular fraction (SVF) was isolated, cultured and differentiated into mature adipocytes as previously described [16]. Insulin sensitivity of fully differentiated primary adipocytes from iWAT, eWAT and BAT was analyzed after 15 min stimulation with 100 nM insulin and subsequent Western blot analysis as previously described [14]. Primary antibodies were the following: phospho-AKT and AKT (CST #4060 and CST #4691, both from Cell Signaling Technologies, Danvers, MA, USA), cytochrome c (136F3) (CST #4280), a-tubulin (CST #2144), OxPhos rodent (#45-8099 ThermoFisher, Waltham, MA, USA), UCP1 (ab10983), b-actin (A2066, Sigma Aldrich, St. Louis, MO, USA). HRP-coupled secondary antibodies (CST#7074). Chemiluminescence was detected using the G:BOX Chemi XX9 documentation system with GeneTools analysis software (Syngene, Cambridge, UK).

### 2.6. Proteomics of AT Depots

Formalin-fixed, paraffin embedded specimens of iWAT and eWAT depots of control or AT*Klk7^−/−^* mice were processed [27] and liquid chromatography tandem mass spectrometry (LC-MS/MS) was performed as described [28]. Each group consisted of four biological replicates. Data analysis was performed with MaxQuant 1.6.12, using tryptic specificity with up to two missed cleavages, fixed cysteine carbamidomethylation, no variable modifications, and a mouse reference proteome sequence file downloaded from EMBL-EBI on 4th February 2020. Statistical analysis of quantitative proteomic data was performed using linear models for microarray analysis (limma) [29,30]. Proteins with an FDR-adjusted *p*-value < 0.05 and a quantitative alteration of >50% were considered to be significantly affected (Supplementary Tables S2–S5). Proteins were defined as exclusively expressed, whenever they were detected in N ≤ 1 samples of group A and N ≥ 3 samples of group B for the respective comparisons. For a broad over-representation analysis, proteins with an unadjusted *p*-value < 0.01 irrespective of fold change were considered, together with proteins exclusively detected in either control or AT*KLK7^−/−^* mice. Over-representation analysis was performed using the R topGO package (Version 2.42.0) [31] and gene ontology (GO) annotation. A broad gene list enrichment analysis for pathways was performed using Enrichr (www.amppharm.mssm.edu/Enrichr) [32,33].

### 2.7. Thyroid Gland Analysis

Thyroid tissue sampling and cryo-sectioning, biochemical analyses, indirect immunofluorescence and imaging as well as thyroid phenotyping by automated image analysis were performed as published [34,35,36]. The thyroid gland was removed, and either snap-frozen in liquid nitrogen (biochemistry), or fixed in 4% PFA in 200 mM HEPES, pH 7.4 (morphology). Cryo-preservation was carried out by incubating the thyroid tissue in Tissue Freezing Medium (Jung, through Leica Microsystems, Wetzlar, Germany) for 24 h, before tissue was frozen in the gas phase of liquid nitrogen and stored at −80 °C until sectioning. Thyroid tissue samples were homogenized and lysed in ice-cold PBS (68 mM NaCl, 63.2 mM Na_2_HPO_4_, 11.7 mM NaH_2_PO_4_, pH 7.2), containing 0.5% Triton X-100, 1 mM EDTA, and protease inhibitors (0.2 μg/mL aprotinin, Sigma-Aldrich (St. Louis, MI, USA), 1153; EDTA; 10 μM E-64, Enzo Life Science, Farmingdale, NY, USA, ALX 260007-M005; 1 μM pepstatin A, Sigma-Aldrich, 77170).

### 2.8. Statistical Analyses

Statistical analyses were performed using GraphPad Prism 7.0. Data are presented as means ± SEM. Differences between groups were analyzed as indicated in the figure legends. Sample sizes for every figure or table are presented in Supplementary Table S6.

## 3. Results

### 3.1. Generation of Klk7^−/−^ Mice

Constitutive *Klk7* knockout was achieved by deleting exons two to five of the *Klk7* gene as previously described [16]. Heterozygous genetic deletion generates a *Cre* PCR product of a 100 bp as well as 276 bp (control) and 402 bp (floxed) instead of a 276 bp band found in control animals. Homozygous *Klk7^−/−^* animals showed a 100 bp *Cre* PCR product (Supplementary Figure S1A). *Klk7^−/−^* mice are fertile and were obtained with the expected Mendelian frequency. Using PCR analysis of genomic DNA, we confirmed *Klk7* gene disruption (Supplementary Figure S1B).

### 3.2. Body Weight Gain and Body Fat Mass are Markedly Reduced in Klk7^−/−^ Mice

**Figure 1:**
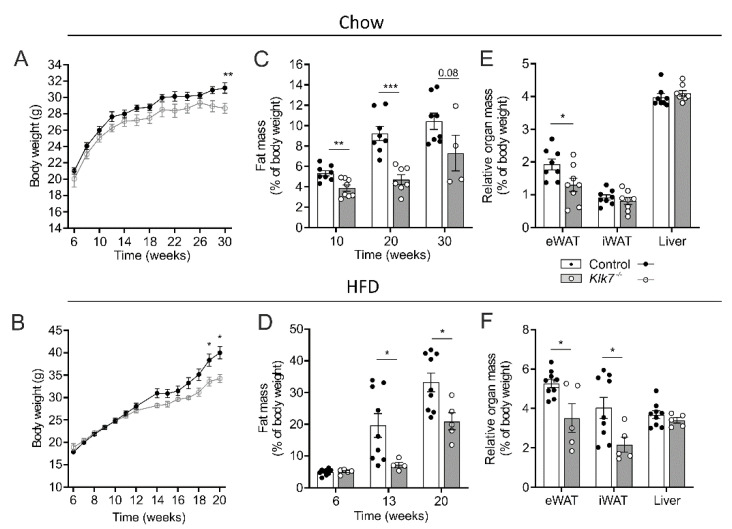
Body weight development and body composition of *Klk7^−/−^* deficient mice under chow and high fat diet. (**A**,**B**) Body weight gain under chow and HFD for *Klk7^−/−^* (*n* = 8/5 chow/HFD) and control mice (*n* = 8/9 chow/HFD). Weight gain is significantly reduced for *Klk7^−/−^* mice under both diets. (**C**,**D**) MRI scans reveal a significant reduction in total fat mass (presented as % of body weight) for *Klk7^−/−^* mice beginning at an age of 10 weeks and independent of the diet. (**E**,**F**) Determination of relative organ weights show that *Klk7^−/−^* mice under chow diet exhibit decreased eWAT fat mass compared to chow-fed control mice, while HFD-fed *Klk7^−/−^* mice have both reduced eWAT and iWAT fat mass compared to obese controls. Data are presented as means ± SEM; significance was tested by two-way ANOVA with correction for multiple testing (Sidak; **A**,**B**) or multiple *t*-tests with correction for multiple testing (Holm–Sidak; **C**–**F**); * *p* < 0.05; ** *p* < 0.01; *** *p* < 0.001.

*Klk7^−/−^* mice gained significantly less body weight than control littermates upon both chow and high fat diet, starting at an age of 14 weeks (Figure 1A,B, Table 1). Lower body weight of *Klk7^−/−^* mice was accompanied by a ~40% significant reduction of total fat mass under HFD (Figure 1C,D). In chow-fed mice, relative eWAT fat mass was significantly reduced, and HFD leads to both, a significant reduction in eWAT and iWAT fat mass in *Klk7^−/−^* vs. control mice (Figure 1E,F). Lean body mass was not altered in *Klk7^−/−^* mice (Supplementary Figure S2). Similarly, liver weight and liver glycogen content were not different (Figure 1E and Supplementary Figure S3).

**Figure 2:**
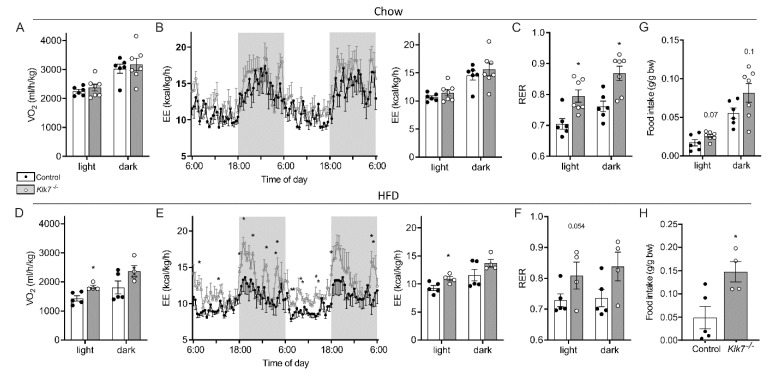
Indirect calorimetry of *Klk7^−/−^* mice under chow and high fat diet. (**A**,**D**) Oxygen consumption (VO_2_ in mL/kg/h, means during light and dark phase), (**B**,**E**) energy expenditure (EE in kcal/kg/h; left panel: time course over 48 h; right panel: means during light and dark phase) and (**C**,**F**) respiratory exchange ratio (RER, as means during light and dark phase) were recorded for a period of 60 h in control and *Klk7^−/−^* mice fed chow diet (**A**–**C**) or HFD (**D**–**F**). (**G**) Food intake (g/g body weight) during light and dark phase over a period of 60 h in mice fed a chow diet. (**H**) Food intake (g/g body weight) per day over a period of 60 h in HFD-fed mice. Data are presented as means ± SEM; significance was tested by two-way ANOVA and Fisher’s LSD test (**B**,**E**) or multiple *t*-tests without correction for multiple testing (summarized data in **A**–**F**) or Student’s *t*-test (**G**–**H**).* *p* < 0.05.

Energy expenditure was assessed using indirect calorimetry (Figure 2). While there was no change in oxygen consumption (Figure 2A) and total energy expenditure (EE, Figure 2B) in chow-fed animals, *Klk7^−/−^* mice under HFD showed significantly higher oxygen consumption (Figure 2D) and EE during the light phase compared to controls (*p* < 0.05; Figure 2E). Locomotor activity was not affected by *Klk7^−/−^*. Interestingly, *Klk7^−/−^* exhibited a preference for carbohydrate oxidation reflected by significantly higher respiratory exchange ratios (RER) compared to controls (Figure 2C,F). As observed in AT*Klk7^−/−^* mice [16], food intake was higher in *Klk7^−/−^* mice, both under chow and HFD (Figure 2G,H).

### 3.3. Consequences of Klk7^−/−^ on Adipose Tissue

**Figure 3:**
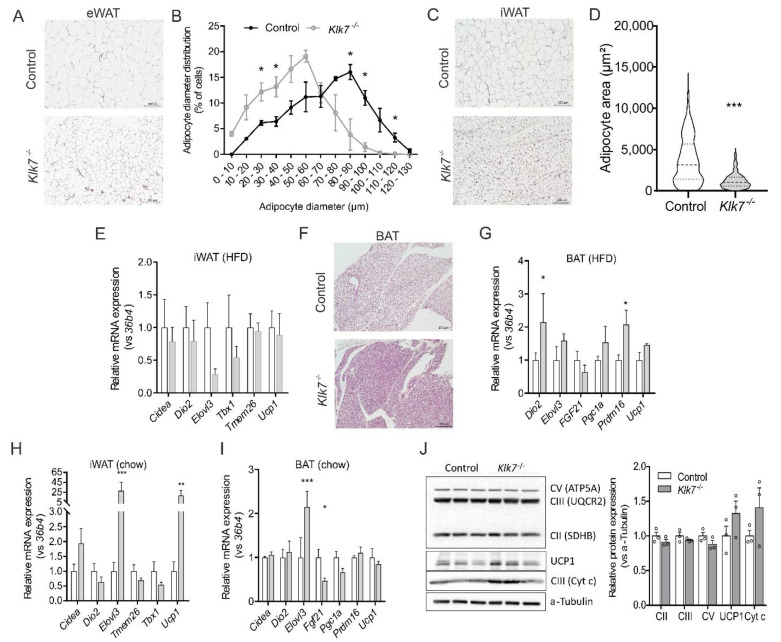
WAT and BAT histology, adipocyte hypertrophy and browning in HFD-fed *Klk7^−/−^* mice. Histological analyses (Hematoxylin and Eosin staining) of epigonadal WAT (**A**) and inguinal WAT (**C**) show highly reduced adipocyte hypertrophy in *Klk7^−/−^* mice with smaller adipocyte diameter (shown for eWAT in (**B**)) and adipocyte area (shown for iWAT in (**D**)). (**E**) Expression of thermogenic genes in iWAT of HFD-fed *Klk7^−/−^* mice is not changed. (**F**) BAT whitening after HFD feeding in less pronounced in *Klk7^−/−^* mice and genes related to BAT activity (**G**) such as *Dio2* and *Prdm16* are higher expressed in *Klk7^−/−^* mice compared to controls. (**H**,**I**) Expression of genes related to thermogenesis and WAT-browning in iWAT and BAT of chow-fed mice shows increased expression of *Elovl3* in both AT depots and highly induced *Ucp1* expression in iWAT of *Klk7^−/−^* mice. (**J**) Western blot analysis of mitochondrial proteins and UCP1 in differentiated primary brown adipocytes from *Klk7^−/−^* mice. Data are presented as means ± SEM; significance was tested by *t*-tests (**D**) or multiple *t*-tests with correction for multiple testing; * *p* < 0.05, ** *p* < 0.01, *** *p* < 0.001.

We performed adipocyte size distribution measurements and AT histology analyses. Expression of genes involved in thermogenesis (*Ucp1, Cidea, Dio2, Elovl3, Fgf21, Pgc1a, Prdm16*) and WAT browning (*Tmem26, Tbx1*) were analyzed in BAT, iWAT and eWAT. In HFD-fed mice, adipocytes were significantly smaller in eWAT (Figure 3A,B) and iWAT (Figure 3C,D) of *Klk7^−/−^* mice compared to controls. There were no differences in the expression of thermogenic genes (Figure 3E). Macrophage infiltration into eWAT of HFD-fed *Klk7^−/−^* mice was diminished, with more M2 and significantly less M1 polarized AT macrophages in the AT samples analyzed (Supplementary Figure S4). In *Klk7^−/−^* mice, HFD-induced whitening of BAT was reduced (Figure 3F) in parallel to increased expression of *Dio2* and *Prdm16* (Figure 3G). In chow-fed *Klk7^−/−^* mice, gene and protein expression related to BAT activity, mitochondrial activity and WAT browning were significantly higher compared to controls (Figure 3I,J).

### 3.4. Klk7^−/−^ Mice Exhibit Lower Body Temperature and Altered Circulating T3

**Figure 4:**
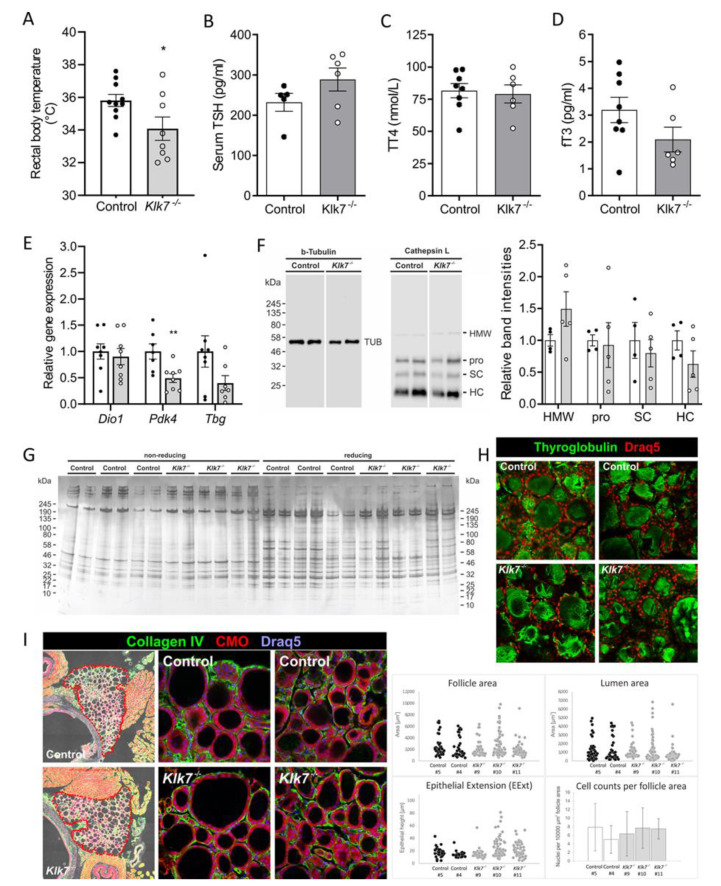
Body temperature, thyroid hormone levels and thyroid gland characterization of chow-fed *Klk7*^−/−^ mice. (**A**) Core body temperature was significantly lower in *Klk7^−/−^* mice (*n* = 10/8, controls and *Klk7^−/−^*). (**B**–**D**) Circulating total serum thyroid-stimulating hormone (TSH), thyroxine (TT4) and free triiodothyronine (fT3) levels were measured. fT3 levels were reduced in *Klk7^−/−^* mice (*n* = 8/6). (**E**) Expression of thyroid responsive genes *Dio1, Pdk4* and *Tbg* in liver of *Klk7^−/−^* and control mice (*n* = 7/8). Expression of *Pdk4* is lower and *Tgb* is significantly reduced. (**F**) Representative immunoblot analysis of cathepsin L expression (pro- (pro), single-chain (SC), heavy chain (HC)) and high molecular weight (HMW)) as well as tubulin (TUB) in thyroid tissue of control and *Klk7^−/−^* mice. Densitometric quantification is presented on the right and revealed no significant changes. (**G**) Thyroid proteins were separated by reducing and non-reducing SDS-PAGE and analyzed after silver-staining, revealing similar banding patterns for both genotypes. Western blot analysis of thyroglobulin expression in thyroid tissue of control and *Klk7^−/−^* mice did not show differences, as did (**H**) immunostaining of thyroglobulin in tissue sections. (**I**) Immunostaining of collagen IV and staining of cytoplasm with CellMask™ Orange (CMO) and nuclei using Draq5™ of thyroid tissue from control and *Klk7^−/−^* mice (left panels). Follicle size and distribution is similar, with larger follicles in the periphery (middle panels) and smaller follicles in the thyroid lobes’ center (right panels). Follicle area, lumen area, epithelial extensions, and cell counts per follicle area (right panels, respectively) appear comparable, again supporting unaltered thyroglobulin synthesis, deposition and solubilization in *Klk7^−/−^* mice. Data are presented as means ± SEM; significance was tested by Student’s t-test (**A**–**D**), two-way ANOVA with correction for multiple testing (Tukey; (**E**)); * *p* < 0.05, ** *p* < 0.01.

Thyroid hormones are major physiological regulators of metabolism and AT function. Surprisingly, body temperature of chow-fed *Klk7^−/−^* was significantly lower compared to control mice (Figure 4A). As multiple kallikrein proteases including KLK7 are expressed in thyroid tissue [37], we investigated potential effects of *Klk7* deficiency on thyroid hormone generation, circulating thyroid stimulating hormone (TSH), total T4 (TT4), and free T3 (fT3) concentrations. While TSH and TT4 remained unchanged, fT3 serum levels were in tendency lower in *Klk7^−/−^* compared to control mice (Figure 4B–D). In the liver, expression of thyroid hormone responsive genes *Pdk4* and thyroxine-binding globulin (*Tbg)* were decreased, while deiodinase 1 (*Dio1*) expression was not altered (Figure 4E). Expression of thyroglobulin solubilizing and degrading enzymes were evaluated in thyroid lysates of *Klk7^−/−^* mice, but protein amounts of aspartic cathepsin D as well as the cysteine proteases cathepsins B and L were not different from controls (Figure 4F and Supplementary Figure S5). In addition, thyroid protein analyses using gel-electrophoresis or immunostaining did not indicate genotype-related abnormalities (Figure 4G,H). Finally, thyroid histology and phenotyping revealed that thyroid follicle size and distribution is similar in control and *Klk7^−/−^* mice (Figure 4I).

### 3.5. Ablation of Klk7 Does Not Affect Circulating Vaspin and Chemerin

*Klk7^−/−^* mice do not develop abnormalities of circulating parameters, except for a 15% increase in FFAs under HFD (Table 1). Importantly, chemerin and vaspin serum concentrations were not different between *Klk7^−/−^* and control mice (Table 1). In proteome analyses, chemerin (RARRES2) was only detected in iWAT of HFD-fed *Klk7^−/−^* mice (Supplementary Table S3).

### 3.6. Klk7^−/−^ Mice Exhibit Increased Sensitivity to Exogenous Insulin

**Figure 5:**
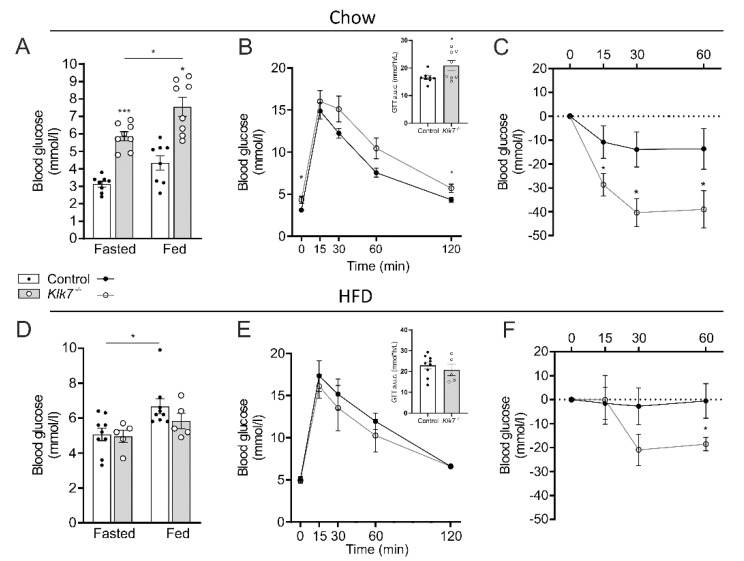
Glucose metabolism/homeostasis and insulin sensitivity in *Klk7^−/−^* mice are contrastingly affected under chow and high fat diet. (**A**) Fasted and fed blood glucose levels of chow-fed *Klk7^−/−^* mice were significantly higher than in controls (*n* = 8/8, controls and *Klk7^−/−^*). (**B**) Glucose tolerance tests (GTT) and are under the curve (a.u.c., presented in insert) in fasted chow-fed mice reveal impaired glucose tolerance for *Klk7^−/−^* mice (n = 8/8). (**C**) Insulin tolerance tests (ITT) in fed mice show significantly higher sensitivity to exogenous insulin for *Klk7^−/−^* mice compared to controls (*n* = 8/8). (**D**) Fasted and fed blood glucose levels of obese *Klk7^−/−^* and control mice are not different (*n* = 9/5). (**E**) GTT and a.u.c. (insert) in fasted obese mice are not affected by the genotype (*n* = 9/5). (**F**) ITT in fed obese animals indicate retained insulin sensitivity in obesity for *Klk7^−/−^* mice (*n* = 9/5). Data are presented as means ± SEM; significance was tested by one-way ANOVA with correction for multiple testing (Tukey; (**A**,**D**)), Student’s *t*-test ((**B**,**E**), a.u.c data), or by two-way ANOVA and Fisher’s LSD test (**B**,**C**,**E**,**F**); * *p* < 0.05, *** *p* < 0.001.

Despite the leaner phenotype, chow-fed *Klk7^−/−^* mice have ~80% higher fasted (*p* < 0.001) and fed (*p* < 0.05) glucose concentrations (Figure 5A). Moreover, *Klk7^−/−^* mice exhibit impaired glucose tolerance, with significantly higher areas under the curve of the GTT in comparison to control animals (*p* < 0.05; Figure 5B). On the other hand, ITTs revealed a pronounced higher sensitivity of *Klk7^−/−^* mice to exogenous insulin compared to controls (Figure 5C).

**Figure 6:**
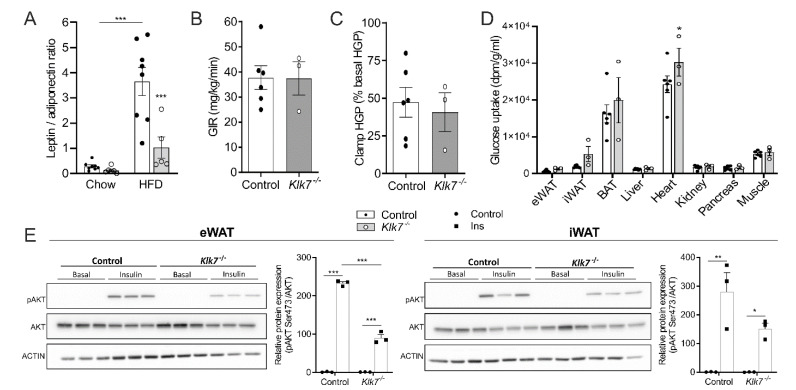
Whole body and tissue insulin sensitivity in chow-fed *Klk7^−/−^* mice. (**A**) Leptin to adiponectin ratios for chow and HFD fed mice as proxy for adipose tissue function indicate improved metabolic state/health in lean and obese *Klk7^−/−^* mice compared to controls (chow *n* = 8/8, HFD *n* = 8/5, controls and *Klk7^−/−^*). (**B**) Glucose infusion rate (GIR in mg/kg/min) during clamp was not different in chow-fed *KLK7^−/−^* mice compared to controls mice (*n* = 6/3). (**C**) Hepatic glucose production (HGP) during clamp (as % from basal HGP) is not affected by the genotype (*n* = 6/3). (**D**) Tissue glucose uptake (dpm/g/mL) is significantly higher in hearts of *Klk7^−/−^* mice compared to controls (*n* = 6/3). (**E**) Insulin-induced AKT phosphorylation analyzed by Western blot in differentiated primary adipocytes from eWAT (left) and iWAT (right) from three animals per genotype. Densitometric quantification of bands is presented next to the blots. Phosphorylation of AKT by insulin is significantly dampened in all primary adipocytes from *Klk7^−/−^* mice. Data are presented as means ± SEM; significance was tested by Student’s *t*-test (**A**–**C**), uncorrected multiple *t*-tests (**D**) or two-way ANOVA with correction for multiple testing (Tukey; (**E**)); * *p* < 0.05, ** *p* < 0.01, *** *p* < 0.001.

In contrast to chow-fed animals, fasted and fed blood glucose levels were not significantly different between *KLK7^−/−^* and control mice in response to HFD (Figure 5D). Despite the leaner phenotype of HFD-fed *KLK7^−/−^* mice, glucose tolerance was not different between the genotypes (Figure 5E). In contrast to *KLK7^−/−^* mice, HFD-fed controls almost lost responsiveness to exogenous insulin (Figure 5F). The leptin to adiponectin ratio and MCP-1 levels were significantly lower in both, chow- and HFD-fed *KLK7^−/−^* mice compared to controls (Figure 6A, Table 1).

Interestingly, in chow-fed mice glucose infusion rate (GIR) during hyperinsulinemic-euglycemic clamp studies was not different between *KLK7^−/−^* and controls (Figure 6B). Hepatic glucose production (HGP) and insulin-induced suppression of HGP were indistinguishable between *KLK7^−/−^* and control animals (Figure 6C). At the organ level, we measured significantly higher uptake of glucose into the heart of *KLK7^−/−^* mice (Figure 6D). Fully differentiated primary adipocytes from eWAT and iWAT of *KLK7^−/−^* mice showed significantly lower insulin-induced AKT activation in Western blot analyses (Figure 6E).

### 3.7. Klk7^−/−^ Mice Have Depot-Specific Protein Expression Signatures in AT

**Figure 7:**
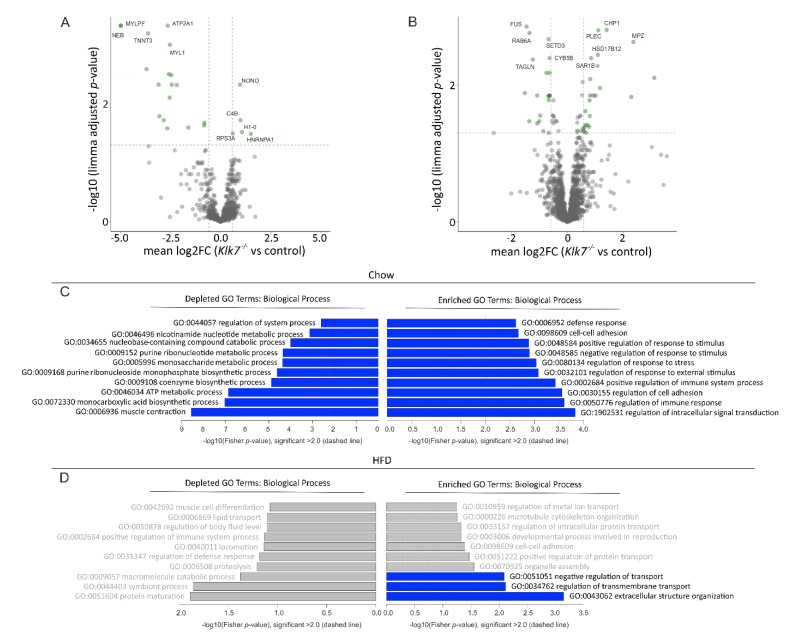
Analysis of proteome expression changes induced in iWAT of AT-specific *Klk7^−/−^* mice. (**A**,**B**) Volcano plots of differentially expressed proteins in iWAT of AT*Klk7^−/−^* mice under chow (**A**) and HFD (**B**) conditions. Protein expression data in the volcano plot are displayed as log2 fold change (FC) versus the log10 of the limma adjusted *p*-value. Proteins with significant differences in expression compared to controls are shown in green. Thresholds are shown as dashed lines. The top five differentially expressed genes (sorted by log-odds) are labelled with corresponding gene symbols. (**C**,**D**) Enriched GO terms related to biological processes in chow- (upper panel) and HFD-fed (lower panel) animals. Vertical bars represent the GO terms; the horizontal axis indicates −log10 (*p*-value). Significantly enriched GO terms are labelled in blue.

As we observed major alterations in AT of *Klk7^−/−^* mice, we aimed to unravel changes in protein expression that may contribute to improved AT function. Therefore, we performed label-free quantitative proteome analyses in eWAT and iWAT depots of adipose-specific *Klk7^−/−^* knockout mice (AT*Klk7^−/−^*) and control mice fed either chow or HFD. First, we compared expression of proteins in eWAT and iWAT between the HFD and chow diet group in controls only. Proteins over- and exclusively expressed in HFD versus chow fed mice were considered as markers of obesity. As shown in Supplementary Table S2, pathways related to PPAR signaling, fatty acid, glucose and cholesterol metabolism were increased in HFD induced obesity. We then focused on the impact of *Klk7* deletion in AT on proteome composition in AT*Klk7^−/−^* mice. Significantly affected proteins were only identified in iWAT (23 under chow and 59 under HFD conditions) (Figure 7A,B). The number of proteins exclusively detected in AT samples was higher in iWAT (52 under chow and 66 under HFD) than in eWAT (8 under chow and 21 under HFD). These data indicate a more prominent role of *Klk7* in subcutaneous AT. There was little overlap between identified proteins in chow and HFD conditions (Supplementary Tables S3 and S4).

Interestingly, we observed a striking accumulation of exclusively detected proteins in iWAT of chow-fed AT*Klk7^−/−^* mice (25 out of 52, Supplementary Table S4). This was accompanied by a number of significantly downregulated markers, thereof multiple tight junction proteins (MYLPF, MYH2, ACTN3, NEB). Pathway analysis revealed enrichment of proteins involved in ER processing of proteins, the phagosome, in amino and nucleotide sugar metabolism as well as PPAR signaling and sucrose metabolism to be affected by loss of *Klk7* in AT. GO term analysis indicated upregulation of proteins involved in the regulation of immune responses as well as intracellular signal transduction and cell adhesion (Figure 7C). Repressed pathways were related to cGMP-PKG as well as calcium signaling (Supplementary Table S5) and proteins related to GO terms of muscle contraction, ATP metabolism and monocarboxylic acid metabolism were significantly repressed in iWAT of chow-fed AT*Klk7^−/−^* mice compared to controls (Figure 7C).

In iWAT of HFD-fed animals, COL3A1, CD163, NCK1, HFE, DNAJC3 and AGT were downregulated AT*Klk7^−/−^* mice compared to controls. KEGG pathway analysis revealed a highly significant induction of metabolic proteins in iWAT of obese AT*Klk7^−/−^* mice that are involved in fatty acid elongation and degradation (Supplementary Table S5). GO terms enrichment revealed significant reduction of proteins involved in extracellular structure organization and regulation of transport (Figure 7D). We compared our data to a dataset comprising potential proteolytic substrates of KLK7 identified by degradome analyses [38]. Overall, putative KLK7 substrates (~20% of differentially expressed proteins) were only observed in iWAT of HFD fed animals, independent of the genotype (Supplementary Tables S3 and S4). Our analyses corroborate a more pronounced impact of diet over *KLK7* genotype on AT proteome. In subcutaneous AT, KLK7 seems to play an important role in the regulation of immune response and metabolism.

## 4. Discussion

We tested the hypothesis that ablation of *Klk7* at the whole body level may cause a metabolically “healthier” phenotype that is distinct from that observed in AT*Klk7^−/−^* mice.

A key finding of the study is that whole-body deletion of *Klk7* resembles the phenotype of AT-specific KO in many aspects. However, beyond the expected growth curve differences from AT*Klk7^−/−^* mice [16], *Klk7^−/−^* mice exhibit a leaner phenotype with aging. Body weight differences between *Klk7^−/−^* and control mice became significant under chow fed conditions at an age of 30 weeks. Both under chow and HFD, body fat mass was significantly lower in *Klk7^−/−^* compared to control mice, whereas lean body mass or organ weights were not affected by *Klk7* ablation.

In contrast to AT*Klk7^−/−^*, *Klk7^−/−^* mice exhibit a generalized fat mass reduction suggesting an additional systemic role of *Klk7* reduction beyond AT-specific effects on adipogenesis or expandability. Counter-intuitively to lower fat mass and preserved lean mass, *Klk7^−/−^* mice had higher food intake than controls. This may represent an (not entirely sufficient) attempt to compensate for higher energy expenditure of *Klk7^−/−^* compared to control mice as found in metabolic chamber experiments. In addition, lower fat mass was accompanied by lower circulating leptin, which may have contributed to higher food intake of *Klk7^−/−^* mice. However, based on our data, we are not able to distinguish potential causality chains whether effects of *Klk7* ablation primarily cause activation of energy metabolism or affect central regulation of food intake. Lower leptin levels, a higher RER, absence of adipocyte hypertrophy and increased thermogenic gene expression may additionally suggest AT browning as another mechanism explaining the leaner phenotype and higher energy expenditure of *Klk7^−/−^* mice. As result of lower fat mass and/or healthier AT, we find circulating adipokine differences between the genotypes with lower levels of systemic inflammatory markers (e.g., MCP1) and higher circulating adiponectin. This phenotype was preserved under HFD conditions and mostly reflects the phenotype of AT*Klk7^−/−^* mice [16].

Thyroid hormones are major physiological regulators of metabolism [39] and partly of AT function [40]. In rats, kallikrein-related peptidases are regulated by thyroid hormones [41]. Although the overall thyroid state remained unaffected in *Klk7^−/−^* mice as indicated by unchanged serum TT4 and TSH levels, fT3 levels were lower in comparison to control animals. Our data suggest that T4-to-T3 conversion rates or T4-inactivation are affected by loss of *Klk7*. However, expression of the outer- and inner-ring deiodinating enzyme Dio1 in the liver was not affected by *Klk7* deficiency, arguing against a liver-mediated change in TH metabolism. To further investigate the role of thyroid hormones on the phenotype of *Klk7^−/−^* mice, we performed detailed biochemical and morphometric analyses. The results indicated unchanged protein amounts of aspartic and cysteine cathepsins, which are the main thyroglobulin processing and degrading enzymes acting in the follicle lumen for solubilization from cross-linked multimers (cathepsins B and L) and in endo-lysosomal degradation of the TH precursor (cathepsins B, D, and L) [34,42,43]. Because thyroglobulin degradation states did not differ between the genotypes, we conclude that thyroglobulin biosynthesis, cross-linking for storage in the follicle lumen and utilization for TH liberation are not altered in *Klk7^−/−^* mice. However, we cannot exclude that decreased fT3 concentrations in the circulation feed back onto thyroid functionality. Hence, a thorough analysis was performed using semi-automated Cell Profiler-based histological examination of thyroid tissue sections [34,35,36], indicating that KLK7 is dispensable for proper thyroid development and function. This is an interesting finding, because the thyroid protease web is characterized by a high degree of redundancy when cysteine peptidases are considered [42,43]. Obviously, serine proteases such as KLK7 are not redundantly co-regulated with thyroglobulin-processing enzymes, and therefore likely play a different role in thyroid physiology.

Based on the phenotype similarities between AT*Klk7^−/−^* and *Klk7^−/−^* mice, we postulate that AT development and metabolism plays an important role in determining the phenotype of *Klk7^−/−^* mice. Compared to iWAT of control mice, we found significant differences in expression of proteins previously connected to AT development, function and expansion, such as COL3A1, CD163, NCK1, HFE, DNAJC3 and AGT [44,45,46,47,48,49]. Thus, the proteome signature of iWAT from AT*Klk7^−/−^* mice reflects a maintained normal AT function under HFD conditions. In addition, regulators of immune response were higher expressed, whereas proteins promoting macrophage-infiltration during the progression of obesity-associated inflammation, such as CD5L [50], were reduced. Together, AT signatures distinctly associated with reduced *Klk7* expression may contribute to lower macrophage infiltration into AT [16].

The (AT-specific) consequences of *Klk7* disruption may be explained by KLK7-related processes in AT including a reduced activation of pro-inflammatory adipokines such as chemerin. Interestingly, chemerin was only detected in iWAT of *Klk7^−/−^* mice with diet induced obesity. We have previously shown that KLK7 activates human chemerin by cleaving the preform into the active chemerin156 [51]. The effects of chemerin on adipocyte insulin sensitivity and glucose uptake are disputed [52,53] and may not alone explain impaired glucose uptake observed in primary adipocytes of *Klk7^−/−^* mice. However, *Klk7^−/−^* mice did not exhibit lower circulating chemerin, suggesting that modulation of chemerin activation by KLK7 does not represent the main mechanism explaining the leaner phenotype. Yet, chemerin is also involved in adipogenesis [54], and this may be in accordance with an increase of iWAT mass [16]. A higher expression of cathepsin L protease in iWAT of *Klk7^−/−^* mice may further contribute to the expansion of this depot, as loss this protease has been shown to affect adipocyte differentiation and lipid accumulation [7].

Both chow and HFD-fed *Klk7^−/−^* mice are more responsive to exogenous insulin. In hyperinsulinemic-euglycemic clamps, we could exclude that higher sensitivity to exogenous insulin was caused by higher insulin sensitivity of *Klk7^−/−^* compared to control mice. Differences in the response to intraperitoneal versus intravenous insulin application could be related to local effects of KLK7 action on insulin degradation. Insulin is cleaved by KLK7 in vitro [55], and inhibition of KLK7 activity is associated with prolonged insulin circulation and action in vivo [15]. One limitation of our study is that we are not able to provide formal proof of the concept that KLK7 modulates insulin action in vivo. Circulating, putatively KLK7-derived insulin fragments or differences in insulin degradation dynamics between *Klk7^−/−^* and control mice would have supported our hypothesis that KLK7-mediated insulin degradation occurs in vivo and contributes to prolonged insulin action. Despite the better response to insulin of *Klk7^−/−^* mice, endogenous insulin does not seem to be sufficient to maintain glucose homeostasis, particularly after a glucose challenge. Under chow fat conditions and despite lower fat mass, *Klk7^−/−^* mice are characterized by higher fasted, fed and GTT glucose levels. These differences were not confirmed under HFD. Because we consider AT as the critical organ of KLK7 action with regard to glucose homeostasis, we further investigated AT insulin sensitivity. Indeed, we find that primary adipocytes from *Klk7^−/−^* mice display lower insulin-induced AKT-phosphorylation compared to adipocytes obtained from controls and may therefore be considered insulin resistant. In contrast, glucose uptake into AT during the steady state of the clamp was not significantly different between *Klk7^−/−^* and control mice. Lower expression levels of AT SERCAs (sarcoplasmic/endoplasmic reticulum calcium ATPases SERCA3 and SERCA1), voltage-dependent anion-selective channel 1 (VDAC1) and ADP/ATP translocase 2 (SLC25A5) detected in our proteome analysis point towards impaired calcium homeostasis and increased cytosolic calcium levels. While we did not analyze hepatic proteome changes, similar changes in hepatic calcium levels could dysregulate liver glucose production via increased gluconeogenesis, thereby contributing to the observed hyperglycemia in chow-fed AT-*Klk7^−/−^* mice.

## 5. Conclusions

Whole body *Klk7* deficiency significantly decreases body weight gain, both age-related and diet-induced. These data confirm and strengthen our recently demonstrated role of *Klk7* in the regulation of body weight, energy metabolism, and obesity-associated adipocyte dysfunction using the AT-specific knockout [16]. Yet, in the constitutional knockout, the protective effects of *Klk7* deficiency against obesity-associated adipose dysfunction and subsequently insulin resistance under HFD can be mainly attributed to lower fat mass (both, of eWAT and iWAT). This is a striking difference to the conditional AT*Klk7^−/−^*, where the protective effects were observed despite no significant differences in HFD-induced weight gain and obesity.

Together with rodent and human studies providing evidence that elevated plasma protease activity is associated with obesity and type 2 diabetes [4,5,6], this argues for the contribution of specific proteases to obesity and related diseases. Our data further suggest KLK7 as a treatment target for these diseases. Over the last years, various small molecule or peptide-based inhibitors of KLK7 have been described [56,57,58,59,60,61,62,63,64,65,66]. Application of these molecules to *Klk7^−/−^* mice may further improve our understanding of KLK7-related or -dependent mechanisms in AT function and obesity development.

## Figures and Tables

**Table 1:** Anthropometric and metabolic parameters in male chow and HFD-fed *Klk7^−/−^* and control mice.

	Chow		HFD	
	Control (n)	*Klk7^−/−^* (n)	Control (n)	*Klk7^−/−^* (n)
Body weight (g)	29.25 ± 0.62 (8)	26.83 ± 0.73 (8) *	39.97 ± 1.38 (9)	34.24 ± 0.89 (5) *
Body length (cm)	9.95 ± 0.73 (8)	9.76 ± 0.11 (8)	9.86 ± 0.09 (9)	10.00 ± 0.10 (5)
Body temp. (°C)			35.81 ± 0.37 (10)	34.09 ± 0.71 (8) *
**Serum lipids**				
Triglycerides (mmol/L)	1.10 ± 0.05 (8)	1.27 ± 0.14 (3)	1.48 ± 0.05 (9)	1.34 ± 0.11 (5)
Cholesterol (mmol/L)	2.32 ± 0.04 (8)	2.33 ± 0.08 (3)	4.60 ± 0.28 (9)	4.14 ± 0.24 (5)
FFA (mmol/L)	1.71 ± 0.10 (8)	2.06 ± 0.23 (3)	2.39 ± 0.05 (9)	2.84 ± 0.20 (5) *
Glycerol (nmol/mL)	670.21 ± 30.49 (8)	722.59 ± 51.94 (7)	889.81 ± 44.83 (7)	848.92 ± 29.95 (2)
**Adipokines**				
Adiponectin (µg/mL)	47.68 ± 1.22 (8)	54.77 ± 1.96 (7) **	48.49 ± 1.02 (8)	49.52 ± 1.39 (5)
Leptin (ng/mL)	13.90 ± 2.69 (8)	6.16 ± 2.41 (7) **	178.59 ± 28.59 (8)	49.45 ± 19.48 (5) *
Vaspin (pg/mL)	65.18 ± 11.15 (7)	58.65 ± 6.18 (8)	122.17 ± 22.57 (4)	136.91 ± 16.43 (4)
Chemerin (ng/mL)	128.52 ± 6.58 (7)	111.56 ± 4.89 (6)	168.69 ± 15.21 (7)	156.29 ± 14.45 (4)
MCP-1 (pg/mL)	215.90 ± 43.91 (7)	141.52 ± 19.81 (8) *	147.35 ± 18.90 (6)	108.55 ± 18.53 (4)

All results are expressed as means ± SEM. Numbers of animals is given in parentheses. Significantly different values are presented in bold, * *p* < 0.05, ** *p* < 0.01.

## Data Availability

All data generated or analyzed during this study are included in the published article and its online Supplementary files. The resources (e.g., *Klk7^−/−^* and AT*Klk7^−/−^* mice) generated and analyzed during the current study are available from the corresponding author upon reasonable request.

## Supplementary Materials

The following files are available online at https://www.mdpi.com/2227-9059/9/2/131/s1. Figure S1: Genotyping of CMV-*Cre*^+^_*Klk7*^fl/fl^ animals, Figure S2: Lean mass in chow and HFD-fed animals, Figure S3: Liver glycogen content in *Klk7^−/−^* mice, Figure S4: Analysis of macrophage polarization in eWAT, Figure S5: Representative Western blot analysis of cathepsin B and D expression, Table S1–S7.

## Abbreviations

AKT	protein kinase B
AT	adipose tissue
a.u.c.	area under the curve
BAT	brown adipose tissue
bp	base pair
bw	body weight
Cidea	cell death inducing DFFA like effector a
CMV	cytomegalovirus
COL3A1	collagen type 3A1
Cre	causes/cyclic recombination
DEG	differentially expressed gene
Dio	deiodinase
DNAJC3	DnaJ heat shock protein family (Hsp40) member C3
EE	energy expenditure
Elovl3	elongation of very long chain fatty acids like 3
ER	endoplasmic reticulum
eWAT	epididymal white adipose tissue
Fc	fragment crystallizable
FC	fold change
FDR	false discovery rate
FFA	free fatty acid
FGF21	fibroblast growth factor 21
fT3	free T3
GIR	glucose infusion rate
GO	gene ontology
GTT	glucose tolerance tests
HC	heavy chain
HFD	high fat diet
HFE	homeostatic iron regulator
HGP	hepatic glucose production
HMW	high molecular weight
HRP	horseraddish peroxidase
i.p.	intraperitoneal
ITT	insulin tolerance tests
iWAT	inguinal white adipose tissue
KEGG	Kyoto Encyclopedia of Genes and Genomes
KLK7	kallikrein-related peptidase 7–protein
*KLK7*	kallikrein-related peptidase 7–human gene
*Klk7*	kallikrein-related peptidase 7–mouse gene
*Klk7^−/−^*	mouse *Klk7* gene knockout
Limma	linear models for microarray analysis
M1	classically activated macrophage
M2	alternatively activated macrophage
MCP-1	monocyte chemotactic protein 1
MRI	magnetic resonance imaging
MYH2	myosin heavy chain 2
MYLPF	myosin light chain
NCK1	NCK adaptor protein 1
NEB	nebulin
PDK4	pyruvat dehydrogenase kinase 4
PE	phycoerythrin
PGC1A	PPAR gamma coactivator 1-alpha
PKG	protein kinase G
PPAR	peroxisome proliferator activated receptor
PRDM16	PR/SET domain 16
RARRES2	retinoic acid receptor responder 2
RER	respiratory exchange ratio
SC	single chain
SERCA	sarcoplasmic/endoplasmic reticulum calcium ATPase
SLC25A5	ADP/ATP translocase 2
SVF	stromal vascular fraction
Tbg	thyroxine-binding globulin
Tbx1	T-box transcription factor 1
TG	triglyceride
TH	thyroid hormone
TMEM26	transmembrane protein 26
TSH	thyroid stimulating hormone
TT4	total T4
TUB	tubulin
UCP1	uncoupling protein 1
Vaspin	visceral adipose tissue-derived serine protease inhibitor
VDAC1	voltage-dependent anion-selective channel 1
