# Supplementary material for: Role of Kallikrein 7 in Body Weight and Fat Mass Regulation
Source: Biomedicines. 2021 Jan 29;9(2):131. doi: 10.3390/biomedicines9020131 (PMC7912635; doi:10.3390/biomedicines9020131)
Supplement: Supplementary file 1 [file biomedicines-09-00131-s001.zip › biomedicines-1083559 Supplementary Data.pdf]

Supplementary Data

Role of Kallikrein 7 in body weight and fat mass regulation

A Kunath, J Weiner, K Krause, M Rheders, A Pejkovska, M Gericke, ML Biniossek, S Dommel, M Kern, A Ribas-Latre, O Schilling, K Brix, M Stumvoll, N Klöting, JT Heiker and M Blüher

Supplementary Figure S1

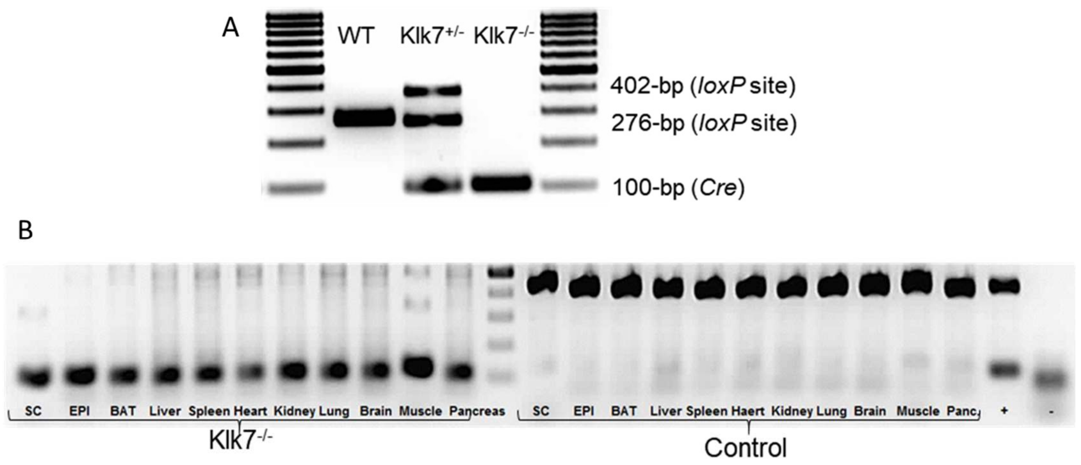

Supplementary Figure S1: Genotyping of CMV-*Cre*$^{+}$_*Klk7*$^{fl/fl}$ animals. (A) Genomic DNA from control mice produced a 276 bp band (lane 2); a 402 bp band (*loxP* site) and a 100 bp (Cre) band was detected in *Klk7*$^{+/-}$ mice (lane 3); only 100-bp (Cre) band was observed in *Klk7*$^{-/-}$ mice (lane 4). (B) Knockdown of different tissues (inguinal (iWAT), epididymal (eWAT) and brown adipose tissue (BAT); liver, spleen, heart, kidney, lung, brain, muscle, pancreas) from control and *Klk7*$^{-/-}$ mice on DNA level.

Supplementary Figure S2

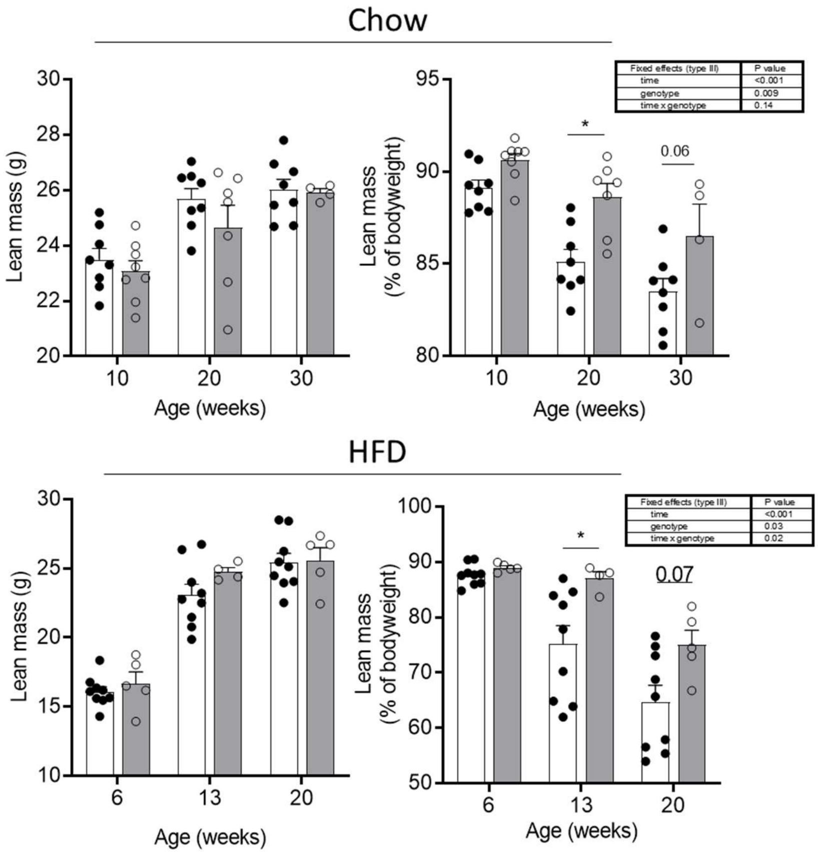

Supplementary Figure S2. Lean mass in chow- (top panel, absolute and relative) and HFD-fed (lower panel, absolute and relative) animals. Data is presented as mean ± SEM. * $p<0.05$, Two-Way ANOVA with Sidak correction for multiple testing.

Supplementary Figure S3

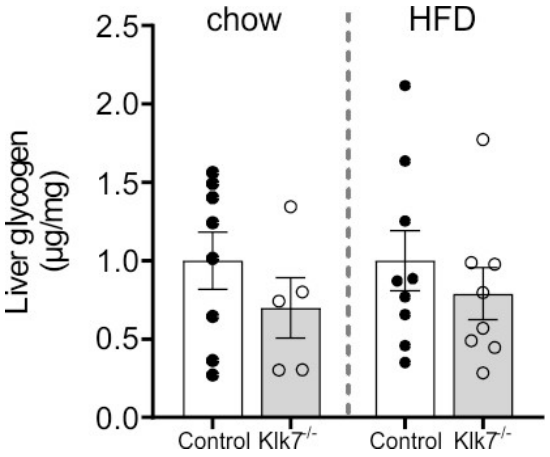

Supplementary Figure S3. Liver glycogen content was not changed in *Klk7*$^{-/-}$ mice under both diets.

Supplementary Figure S4

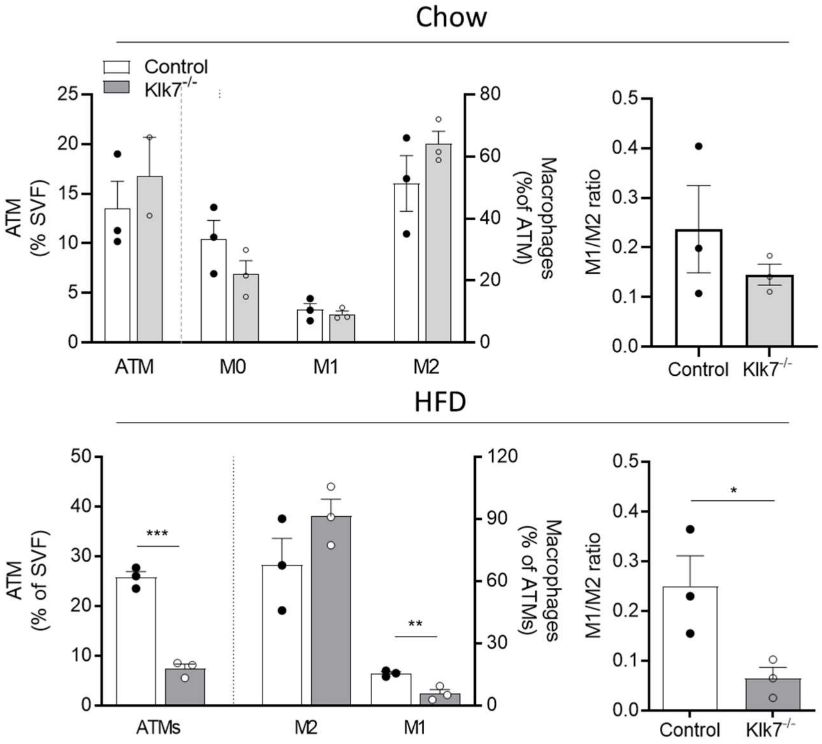

Supplementary Figure S4. Analysis of macrophage polarization in eWAT. Total ATM percentage as well as M0, M1 and M2-polarized macrophages are presented. Furthermore the M1/M2 ratio was calculated for *Klk7*$^{-/-}$ and control mice (n = 3 per genotype). Data are presented as mean ± SEM and differences between genotypes were tested for statistical significance by two-tailed Student's t-test; *P < 0.05, **P < 0.01, ***P < 0.001.

Supplementary Figure S5

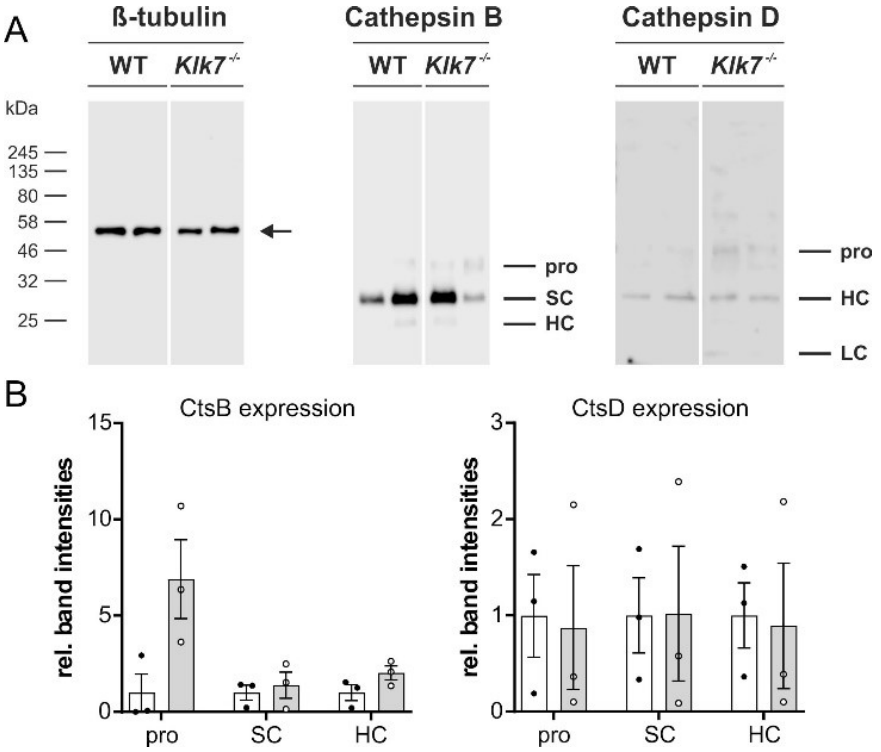

Supplementary Figure S5. Representative Western blot analysis of cathepsin B and D expression (pro- (pro), single-chain (SC), and heavy chain (HC), high molecular weight (HMW) and tubulin (TUB) in thyroid tissue of control and *Klk7*$^{-/-}$ mice. Densitometric quantification is presented on the right and revealed no significant changes.
